# Femtogram-Sensitive Cantilever Platform for Dynamic Graphene Oxide Nanosheet Monitoring

**DOI:** 10.1021/acs.analchem.4c01714

**Published:** 2024-09-09

**Authors:** Pei-Ying Lin, Sheng-Han Cheng, Yu-Chieh Hsu, David E. Beck, Shuchen Hsieh

**Affiliations:** † Department of Chemistry, National Sun Yat-sen University, 70 Lien-Hai Rd., Kaohsiung 80424, Taiwan; ‡ Oxford Instruments Asylum Research, Inc., 7416 Hollister Ave., Santa Barbara, California 93117, United States; § Regenerative Medicine and Cell Therapy Research Center, Kaohsiung Medical University, 100 Shih-Chuan 1st Rd., Kaohsiung 80708, Taiwan; ∥ School of Pharmacy, College of Pharmacy, Kaohsiung Medical University, 100 Shih-Chuan 1st Rd., Kaohsiung 80708, Taiwan; ⊥ Institute of Aquatic Science and Technology, College of Hydrosphere Science, National Kaohsiung University of Science and Technology 142, Haijhuan Rd., Kaohsiung 81157, Taiwan

## Abstract

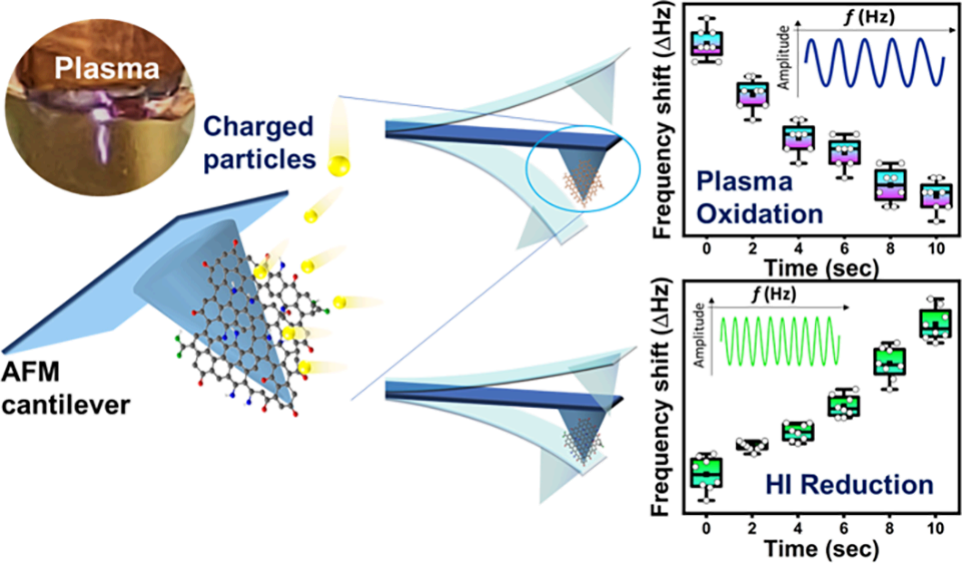

This study introduces a new approach to optimizing graphene oxide (GO) properties using liquid-phase plasma treatment in a microenvironment. Our innovation exploits atomic force microscopy (AFM) cantilever frequency tracking to monitor mass variations in GO, which are indicative of surface oxidation–reduction processes or substituent doping (boron/nitrogen). Complementary in situ Raman spectroscopy has observed D/G band shifts, and X-ray photoelectron spectroscopy (XPS) determined the C/O ratio and B/N doping levels pre- and post-treatment, confirming chemical tuning to GO. We can achieve femtogram-level precision in detecting nanomaterial mass changes by correlating elemental ratios with AFM cantilever frequency measurements. This multifaceted approach not only enhances our understanding of the chemical properties of GO but also establishes a new, versatile method for monitoring, modifying, and optimizing the properties of nanomaterials.

## Introduction

Graphene oxide (GO) is a very promising two-dimensional (2D) carbon-based material due to its versatility and potential use in a wide variety of applications.^1^ GO is the oxidized form of graphene. Its structure, apart from being composed of single-layer carbon atoms arranged in a hexagonal lattice, includes oxygen containing hydroxyl groups (−OH), epoxy groups (C–O–C), and carboxyl groups (−COOH), forming a complex and highly tunable material.^2^ Plasma treatment is a promising method for surface modification of materials. Plasma bombardment of graphene or GO surfaces induces physical lattice distortions and defect formation, altering the carbon/oxygen atomic ratio and structure of these carbon materials.^3^ On the other hand, hydrogen iodide (HI) is widely used as a reducing agent for GO. The effective reduction of GO by HI is attributed to the nucleophilic substitution of epoxy and hydroxyl groups by I– ions. The relatively low bond dissociation energy of C–I bonds facilitates the rapid removal of these oxygen-containing groups.^4,5^ This reduction results in fewer structural defects in the graphene or GO. Moreover, the incorporation of dopant atoms, such as nitrogen (N) or boron (B), offers additional possibilities for tailoring the electrical properties of GO. For example, N-doped GO (N-GO) exhibits n-type electronic properties, while B-doped GO (B-GO) exhibits p-type properties. Doping of GO with either nitrogen or boron can significantly alter the band gap of GO and enhance its suitability for use in electronic,^6^ catalytic,^7^ and electrochemical energy storage^8^ and sensing applications.^9,10^ We hypothesize that the structure of GO changes in its elemental composition as a result of these oxidation or reduction processes, which in turn affects the changes in mass. Therefore, precise control and quantification of the oxygen or dopant levels in GO are essential for tailoring its properties and optimizing performance. Measuring the mass changes of microscopic and nanoscale materials may provide better feedback on the physicochemical reactivity at a fundamental level. Mass changes can be used to determine variations in material composition, reactions, chemical structures, and properties. However, measuring mass changes at the molecular or nanoscale is not trivial. Some existing established analytical methods, such as the quartz crystal microbalance (QCM), high-resolution spectroscopy analysis, and mass spectrometry, have limitations in measuring mass changes due to material selectivity (gas, solid, or liquid state), size (nanoscale), concentration limits, environment (e.g., material photosensitivity), instrument complexity, and more,^11,12^ adding to the challenge and applicability of detection.

Atomic force microscopy (AFM) is an indispensable tool in nanoscience, enabling high-resolution imaging and material property mapping of a wide range of samples under a variety of conditions.^13^ In particular, AFM has been essential in the characterization and optimization of 2D nanomaterials. AFM cantilever frequency analysis has been used for sensing various chemical and biological entities in microelectromechanical resonant sensors.^14,15^ Techniques like QCM have high sensitivity for detecting minute mass changes based on frequency measurements.^16,17^ However, applicability of QCM is limited by larger sample reaction volumes and concentration requirements. These limitations prevent QCM from achieving measurements at smaller scales, which is a persistent challenge for current technology. AFM cantilevers hold promise for achieving higher sensitivity and more precise frequency measurements. Furthermore, AFM can be used to characterize samples in liquid or gas phase and requires only very small sample amounts or concentrations for study. The AFM cantilever frequency shift technique involves monitoring changes in the probe resonance frequency to determine mass changes in the sample under study.

**Figure 1:**
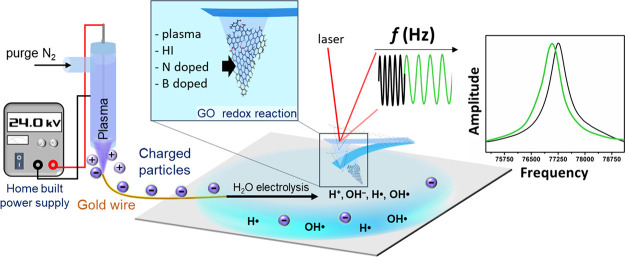
Schematic diagram of GO treatment using a plasma setup.

We hypothesize that the oxidation–reduction treatment of GO with plasma and HI induces mass changes. Additionally, through plasma-mediated chemical tuning, the incorporation of N or B atoms into GO results in induced mass changes. In this study, our aim is to measure the mass changes of GO in the liquid phase after undergoing oxidation–reduction treatment with plasma and HI using the AFM cantilever frequency analysis technique. We also monitored the mass changes of GO doped with boron and nitrogen, as shown in Figure 1. This technique introduces a microscopic and higher-resolution approach for investigating local surface chemical transformations in 2D carbon-based materials.

## Experimental Section

### Graphene Oxide Sheet Synthesis

The synthesis of GO sheets was carried out based on previous experiments.^15^ GO sheets were prepared by self-assembly of an octadecyltrichlorosilane (OTS) film on a silicon wafer. The OTS films were heated in a high-temperature furnace for 30 min at 300 °C, followed by immersion in 1 mL of pure water. Subsequently, the film was rapidly placed in an ultrasonic bath (Delta DC200H, operating at 40 kHz and 200 W) for 2 min to yield GO sheets in the supernatant, which served as the prepared solution for experiments. The oxidation of GO was carried out through plasma treatment, while the reduction reaction involved mixing 10 μL of 5 mM HI with 10 μL of GO. For the doping of GO with nitrogen (N), or boron (B), 1.6 M NH_3_ or 0.8 M H_3_BO_3_ was added separately, and the doping reactions were carried out under plasma conditions.

### Plasma Process

Our method utilizes plasma-induced water electrolysis to generate free radicals for conducting oxidation–reduction reactions of GO. Plasma is continuously generated in the reaction glass tube through a high-voltage power supply, applying a specified voltage of 24 kV, and a nitrogen (N_2_) gas flow rate of 1.5 NL/min before being blown out. We employed a remote liquid-phase plasma process to conduct localized experimental reactions within limited space and avoid disturbances from water in the liquid-phase reaction. In this setup, high-energy plasma is directly applied to a 99.99% pure gold wire with a diameter of 25.4 μm and a resistance of 1.16 Ω/inch, which is in contact with the reaction liquid 10 cm from its end. The gold wire serves as a rapid pathway for charged particles within the plasma, resulting in a high charge and generating a current of 2–3 μA. These charged particles conduct through the gold wire into the water, enabling the oxidation and electrolysis of water and the generation of free radicals. The presence of free radicals in the water is indirectly confirmed through the DPPH analysis method. Subsequently, AFM measurements and analysis are conducted, as shown in Figure 1.

### AFM Cantilever Frequency Analysis

The AFM system used in this study was an MFP-3D AFM, manufactured by Asylum Research, based in Santa Barbara, California. The AC240TS probe from Olympus, Japan, was chosen for all cantilever frequency analyses. Before each probe’s use, the spring constant (*k*) was calibrated using the thermal calibration method, and its relationship is described using eq 1:
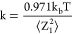
1where *k* represents the spring constant of the cantilever, 0.971 is the first flexural mode correction factor (MCF), *k*_b_ is the Boltzmann constant, ⟨*Z*_1_^2^⟩ is the mean squared thermal deflection, and *T* is the absolute temperature. After calibration, the spring constant for each probe was determined to be 1.9 ± 0.7 nN/nm. The resonance frequency of the cantilever in the liquid phase (H_2_O) was measured as the initial frequency. Next, 20 μL of GO solution was dropped onto the calibrated probe and allowed to sit for 10 min. During this time, GO was adsorbed onto the silicon-based cantilever through hydrogen bonding. The cantilever with adsorbed GO was then transferred to clean DI water 50 μL and was ready for use in various redox treatments and dopant element tests. The resonance frequency of the cantilever was recorded to measure the frequency changes. Each frequency data point was recorded every 2 s. Finally, the GO mass change was calculated from the resonance frequency using the relationship between the cantilever’s resonance frequency and mass, as described in eq 2:^14,18^
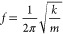
2where *k* is the spring constant, and *m* is the effective mass of the cantilever. The mass change before and after the reaction is represented as Δ*m* = *f*_o_ – *f*. Here, *f*_o_ is the resonance frequency of the cantilever with attached GO before treatment, and *f* is the resonance frequency at different reaction times.

## Results and Discussion

### Gravimetric Detection of GO Redox Based on Resonance Frequency

**Figure 2:**
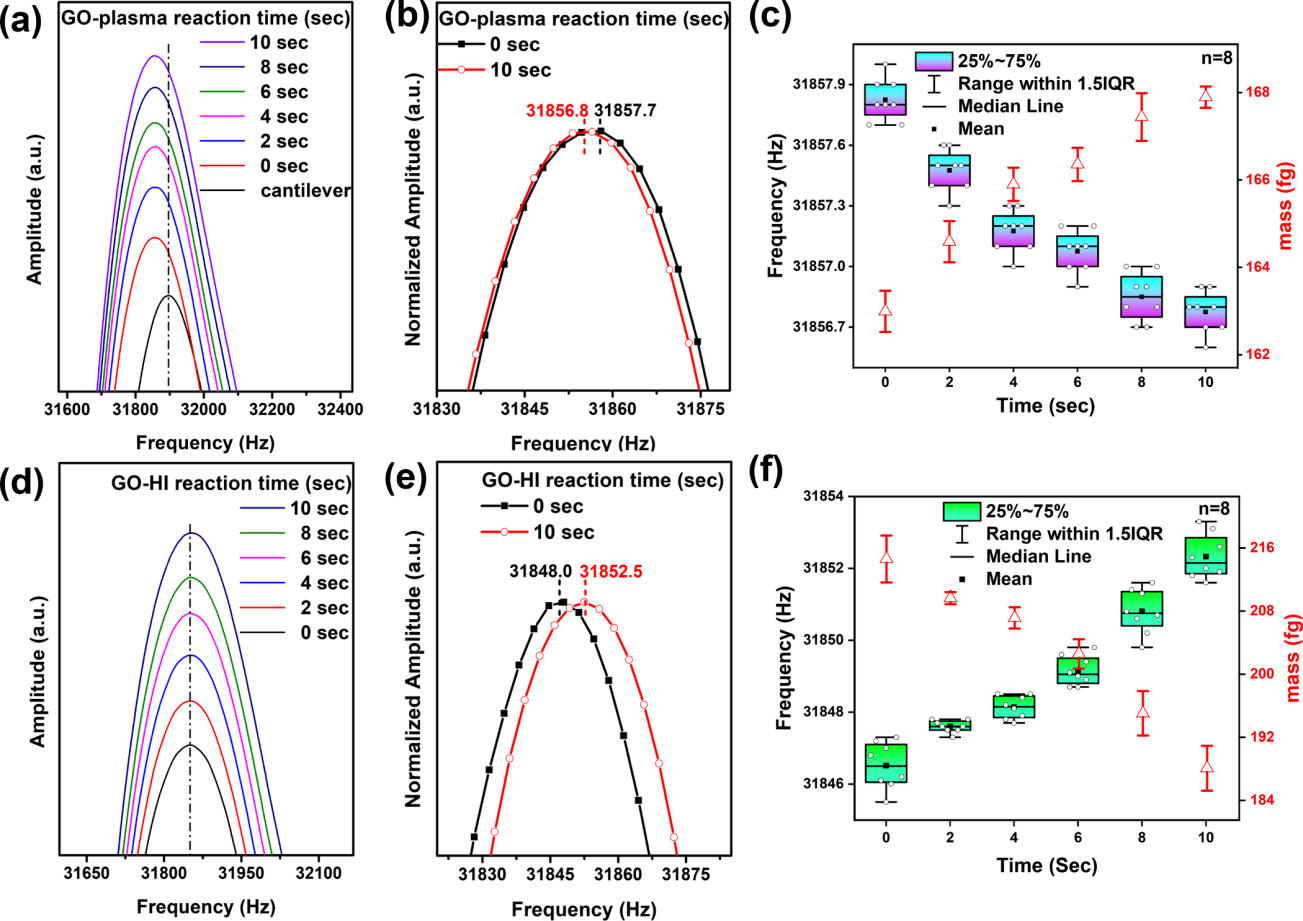
Frequency shifts for cantilever with attached GO sheet as a function of plasma (a–c) or HI (d–f) reaction time. (a) Plasma (0–10 s), (b) comparison between 0 and 10 s plasma treatment, (c) box–whisker plot illustrating the reproducibility testing (*n* = 8) of cantilever frequency changes with different durations of plasma treatment (0–10 s), and the corresponding mass changes (red line). (d) HI (0–10 s), (e) comparison between 0 and 10 s HI treatment, (f) box–whisker plot illustrating the reproducibility testing (*n* = 8) of cantilever frequency changes with different durations of HI treatment (0–10 s) and the corresponding mass changes (red line).

We loaded GO onto the AFM cantilever and monitored the resonance frequency during plasma treatment at different times (0–10 s), as shown in Figure 2. The initial resonance frequency of the cantilever was 31,893.6 ± 7.3 Hz. After loading GO onto the AFM cantilever, the resonance frequency shifted significantly to 31,857.7 ± 0.2 Hz, indicating successful GO loading onto the cantilever (Figure 2a). The resonance frequency consistently shifted to lower frequencies when subjecting GO to plasma treatment for different durations (0–10 s). After 10 s of plasma treatment, the resonance frequency shifted to 31,856.8 ± 0.1 Hz (Figure 2b). The box plot in Figure 2c illustrates the reproducibility testing of cantilever frequency displacement (*n* = 8). The results indicate consistent trends in the cantilever frequency variation for each measurement, demonstrating excellent reproducibility. To validate the accuracy of the GO oxidation and reduction measurements, repeated testing of the AFM cantilever was conducted. Each experiment, consisting of five cycles of alternating 10 s periods of plasma oxidation and HI reduction, was conducted three times to ensure reliability. The results indicate that the frequency of the AFM cantilever exhibited stable mass changes across the five oxidation–reduction cycles. This suggests that the reported mass changes are consistent and reliable (see Figure S1).

Using eq 2, we calculated the mass change of GO before and after plasma treatment, which increased from 163.5 to 167.2 fg (red line in Figure 2c). This mass change is likely due to the oxidation of GO by free radicals in the water,^20,21^ leading to an increase in oxygen-containing functional groups on the GO structure. The presence of free radicals in the water was indirectly confirmed through DPPH tests.^22,23^ After treating pure water with plasma, the free radical concentration in the water was approximately 27.0 μM (relative to the concentration of the vitamin C standard). However, in water containing GO, some of the free radicals reacted with GO, leading to a decrease in free radical concentration to 22.9 μM (relative to the concentration of the vitamin C standard) (see Figure S2).

**Figure 3:**
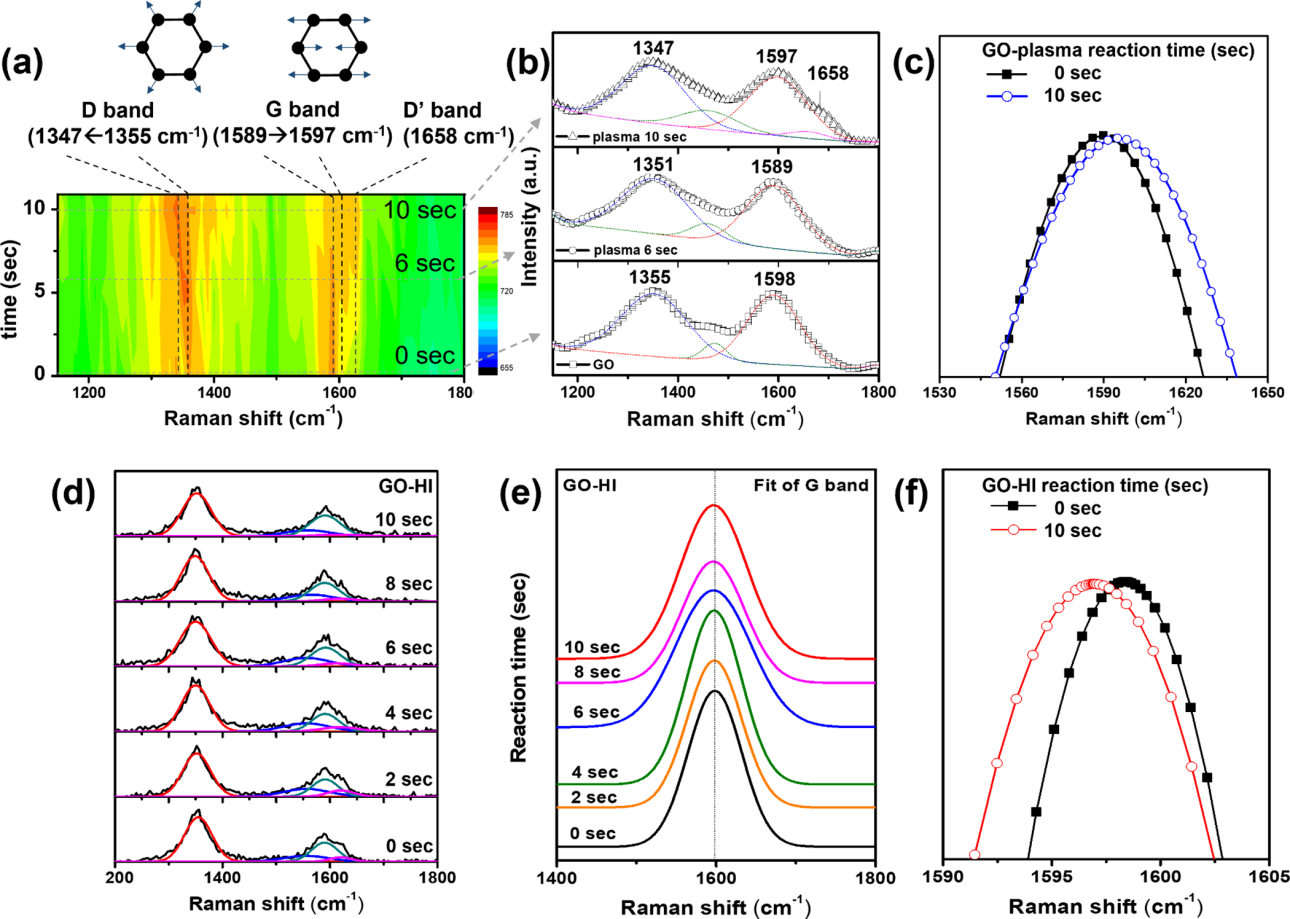
(a) Two-dimensional plot of the Raman signal monitored during 10 s plasma treatment of graphene oxide (GO). (b) Raman spectra of GO at discrete plasma treatment times. (c) Raman G band fit for plasma-treated GO before and after plasma. (d) Raman spectra of GO at discrete HI treatment times. (e) Raman G band fit for discrete HI treatment times. (f) Raman G band fit for HI-treated GO before and after treatment.

To further confirm the oxidation–reduction reactions of GO, we used real-time Raman spectroscopy to monitor the spectral changes of GO during plasma treatment over time. In the 2D plot of time-resolved Raman spectra shown in Figure 3a, typical peak signals of GO sheets were observed at 1355 and 1589 cm^–1^, attributed to the D band and G band, respectively.^24^ The D band represents the noncrystalline phase of GO (associated with defects and disorder in the carbon lattice), while the G band corresponds to vibrational modes in the crystalline phase.^25^ During the plasma treatment, we recorded Raman spectra every 2 s. It was observed that the D band broadened and shifted to 1347 cm^–1^ with increasing treatment time, as shown in Figure 3c. This change can be attributed to the attack of free radicals in the water on GO, leading to the disruption and oxidation of the C–C structures within GO. This process results in defects and deformation in the GO structure, forming sp^3^ carbon atoms, making the structure more loose or irregular.^26^ This also causes a reduction in the size of the sp^2^ domains in GO, leading to a blue shift of the G band to 1598 cm^–1^.^27,28^ Simultaneously, a new peak, the D′ band, appeared at 1658 cm^–1^, which can be attributed to the doping of oxygen functional groups (such as C=O, C–OH, and C–COOH)^29^ (Figure 3b).

Next, we conducted XPS measurements to analyze the elemental composition of GO before and after plasma treatment (see Figure S3). In the C 1s spectrum of the initial GO, four fitted peaks were observed at binding energies of 284, 285.4, 287.2, and 289.2 eV, corresponding to the binding environments of C=C/C–C, C–OH, C=O, and HO–C=O bonds, respectively.

The O 1s spectrum also showed three fitted peaks assigned to C–OH (530.8 eV), C=O (532 eV), and HO–C=O (533.4 eV) oxygen functional groups.^30^ Based on the integrated areas under the curves, the weight percentages of carbon and oxygen elements were determined to be 54.2 and 45.8%, respectively, with a C/O ratio of 1.19 (see Table S1). After plasma treatment, the oxygen proportion increased to 46.0%, while the carbon proportion decreased to 54.0%, resulting in a C/O ratio of 1.17. This shift indicates the oxidation of GO. Further calculations based on the XPS elemental weight percentages and the weight derived from the resonance frequency shift allowed for the precise determination of the atomic counts of carbon and oxygen in GO after plasma treatment, as shown in Table 1. The initial GO had 4.44 × 10^9^ carbon atoms and 3.75 × 10^9^ oxygen atoms, while after plasma treatment, GO had 4.54 × 10^9^ carbon atoms and 3.87 × 10^9^ oxygen atoms.

**Table 1:** Number of Atoms and the C/O Ratio Change in the GO-Treated Reaction

	atom	mass (fg)	number of atoms	ratio
	GO-plasma			
before	C	88.56	4.44 × 10^9^	1
	O	74.70	3.75 × 10^9^	0.843
after	C	90.50	4.54 × 10^9^	1
	O	77.17	3.87 × 10^9^	0.853
	GO-HI			
before	C	112.17	5.63 × 10^9^	1
	O	95.64	4.80 × 10^9^	0.853
after	C	78.32	3.93 × 10^9^	1
	O	63.35	3.18 × 10^9^	0.809
	I	45.47	2.28 × 10^9^	0.581

Next, we used HI to reduce the plasma-treated GO, monitoring the mass change through the resonance frequency of the AFM cantilever, as shown in Figure 2d. The results indicate that as the reaction time between GO and HI increased, the resonance frequency shifted toward higher frequencies, from 31,848.0 ± 1.3 to 31,852.5 ± 3.1 Hz, resulting in a mass change from 214.6 ± 2.9 to 188.1 ± 2.8 fg (red line in Figure 2f). The reaction exhibits excellent reproducibility over multiple repeated tests, demonstrating consistent results in cantilever frequency changes. This mass change can be attributed to the reduction process facilitated by HI under acidic conditions, where HI can remove hydroxide ions via nucleophilic attack. Subsequently, iodide ions act as good leaving groups, readily detaching from the graphene surface to reform carbon–carbon bonds into hexagonal ring structures.^31,32^ This reduces oxygen functional groups on GO and enhances crystallinity (see Figure S4). The FTIR results show pure GO peaks at 1359 and 1780 cm^–1^, corresponding to C–OH bending vibrations and C=O stretching, respectively (see Figure S5).^33^ For GO-plasma, we observed a new peak at 1165 cm^–1^, corresponding to C–O–C stretching. Additionally, the intensity of the C–OH bending vibration signal increased, indicating a higher degree of oxidation. After reduction with HI, we observed a new peak at 473 cm^–1^, attributed to the C–I vibration mode, suggesting that iodine was doped into the GO.^34^ The intensity of the C=O stretching (1780 cm^–1^) and C–OH bending vibration (1435 cm^–1^) signals significantly weakened, indicating the reduction of GO. The C–O stretching observed is likely due to the edge defect cyclic ether contribution.^35^ Furthermore, iodine signals were not observed in the LC-MS analysis when GO was reduced by HI, possibly due to the low iodine content. However, during the HI reduction of GO, we detected *m*/*z* values of 169.03 and 181.17, which could correspond to the fragments [M + H] C_3_H_5_I and C_4_H_5_I, respectively (see Figure S6), resulting from the reduction of GO by iodine. Therefore, the infrared spectroscopy, Raman spectroscopy, and XPS analyses provided more substantial evidence to confirm the outcomes of these reactions.

Raman spectra showed that with increasing reaction time with HI, the G band Raman signal shifted gradually to lower wavenumbers, from 1598 to 1596 cm^–1^, and the D′ band (1658 cm^–1^) signal weakened gradually (Figure 3d–f). Furthermore, XPS analysis of the composition of GO after HI reduction revealed the presence of carbon, oxygen, and iodine elements. The C 1s spectrum showed a new binding energy at 286 eV, attributed to I–C–OH (Figure S3a),^36^ indicating the partial incorporation of iodine into GO. The elemental percentages of carbon, oxygen, and iodine were 41.9, 33.9, and 24.3%, respectively. Through AFM resonance frequency calculations, the atomic counts of carbon, oxygen, and iodine in GO were determined to be 3.93 × 10^9^, 3.18 × 10^9^, and 2.28 × 10^9^, respectively. This result demonstrates that we can accurately monitor the mass changes in the oxidation–reduction of GO using the AFM cantilever’s resonance frequency.

### Gravimetric Detection of N- and B-Doped GO

**Figure 4:**
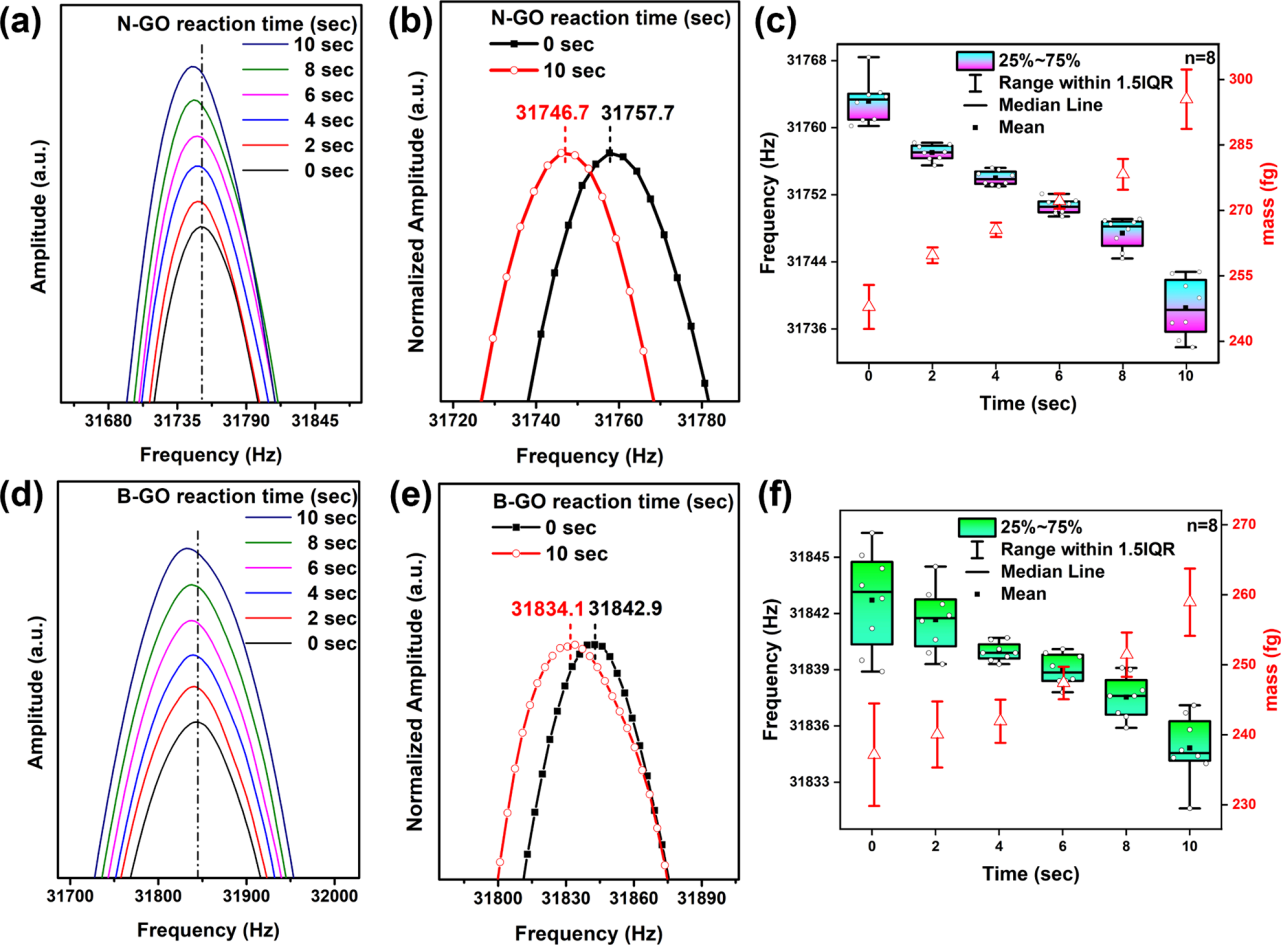
Cantilever frequency change for a probe with an attached GO sheet as a function of nitrogen (a–c) and boron (d–f) doping reaction times. (a) N doping (0–10 s), (b) comparison between 0 and 10 s of N doping, (c) box–whisker plot illustrating the reproducibility testing (*n* = 8) of cantilever frequency changes with different durations of N doping (0–10 s) and the corresponding mass changes (red line). (d) B doping (0–10 s), (e) comparison between 0 and 10 s B doping, (f) box–whisker plot illustrating the reproducibility testing (*n* = 8) of cantilever frequency changes with different durations of B doping (0–10 s) and the corresponding mass changes (red line).

Elemental doping of GO is of great interest due to the structural and electronic modifications that can be created. To establish the broad applicability of the AFM cantilever frequency measurement method, we used boric acid and ammonia through plasma treatment to monitor the mass changes in GO doped with N and B, as shown in Figure 4. The results indicated that during the doping process of N or B elements into GO, the resonance frequency shifted to lower frequencies at 10 s reaction time. As shown in Table S2, the resonance frequency shift for GO-N doping decreased from 31,757.7 ± 3.8 to 31,746.7 ± 2.3 Hz, and the calculated mass change increased from 258.3 ± 7.3 to 279.5 ± 4.5 fg (Figure 4b).

Similarly, for GO-B doping, the resonance frequency shift decreased from 31,842.9 ± 0.2 to 31,834.1 ± 2.5 Hz, resulting in a mass change increased from 236.6 ± 0.5 to 260.9 ± 7.0 fg (Figure 4e). This mass change can be attributed to the doping of N and B, leading to weight variations in GO, as observed in the XPS spectra (see Figure S7). In the C 1s spectrum of N-GO, four fitted signal components were located at 284, 285.6, 286.2, and 287.8 eV, corresponding to C=C/C–C, C–N, C=N, and N–C=O, respectively (see Figure S7a). In contrast to GO, no signal indicative of N was detected. The N 1s spectrum displayed four components at 398.1, 399.4, 401.6, and 403.4 eV (see Figure S7c), corresponding to pyridinic N, pyrrolic N, graphitic N, and oxidized N bonding environments (see Figure S8),^22^ indicating the incorporation of N into GO.

Boron-doped GO exhibited similar results (see Figure S7d–f). The C 1s spectrum showed a new peak at C–B (283.5 eV). In the B 1s spectrum, three components were fitted at 191.1, 192.6, and 193.3 eV, corresponding to BC_3_, BC_2_O, and BCO_2_, respectively, indicating that B forms bonds with GO in these four different forms. This is attributed to the formation of free radicals due to high-energy electron bombardment of boric acid in the plasma. Subsequently, it bonds at the edges of GO to form BCO_2_. Furthermore, B–C single bonds have lower bond energies and are easily cleaved by free radicals compared to single bonds in BC_2_O and BC_3_, leading to the transformation into the more stable BC_2_O and BC_3_ structures (see Figure S9).^37,38^

Moreover, the observation of the Raman peak position shift revealed that the G band blue-shifted with increasing N doping time, indicating lattice distortion and defects resulting from the substitution of nitrogen atoms into the GO lattice (see Figure S10a–c). Literature reports indicate that the distance between adjacent carbon atoms in the C–C plane is 1.42 Å, while the C–N bond length for pyrrolic nitrogen formed on GO is ∼1.37 Å. This implies that in the process of N doping into GO, the formed C–N bond has a shorter length than C–C, resulting in higher energy and compressive strain.^39^ In contrast, for GO-B doped, the C–B bond length is ∼1.56 Å, which is greater than the C–C bond,^40^ indicating lower energy and the formation of tensile strain. Consequently, the G band gradually red-shifted with increasing B doping reaction time (see Figure S10d,e). Based on the AFM resonance frequency mass and XPS elemental ratios (Table 2), we calculated that the numbers of carbon, oxygen, and nitrogen atoms in the GO-N doped were 7.63 × 10^9^, 6.37 × 10^9^, and 1.43 × 10^9^, respectively. For GO-B, the numbers of carbon, oxygen, and boron atoms were 6.74 × 10^9^, 6.34 × 10^9^, and 8.18 × 10^6^, respectively.

**Table 2:** Number of Atoms and the C/O Ratio Change in Different GO N and B Doped

	atom	mass (fg)	number of atoms	ratio
	GO-N doped			
before	C	140.15	7.03 × 10^9^	1
	O	118.20	5.93 × 10^9^	0.843
after	C	152.19	7.63 × 10^9^	1
	O	127.05	6.37 × 10^9^	0.835
	N	0.28	1.43 × 10^7^	0.002
	GO-B doped			
before	C	128.36	6.44 × 10^9^	1
	O	108.26	5.43 × 10^9^	0.843
after	C	134.30	6.74 × 10^9^	1
	O	126.47	6.34 × 10^9^	0.942
	B	0.16	8.18 × 10^6^	0.001

Finally, we monitored the frequency changes of AFM cantilevers loaded with GO during extended oxidation, reduction, or doping reaction times (see Figures S11 and S12). Further piecewise linear regression analysis^41^ showed that the GO mass remained stable as the oxidation or reduction reaction time extended to 12 s (see Figure S11a,b). The slopes during the 0–12 s period for the oxidation and reduction reactions were 1.637 and −0.576, respectively. After 12 s, the slopes were −0.019 and −0.041, respectively, indicating that the mass change of the GO had approached equilibrium in both reactions. Kinetic analysis fitted to the 0–8 s reaction time range indicated that the oxidation and reduction reactions of GO followed a pseudo-first-order kinetic model, with *R*^2^ values of 0.98 and 0.96, respectively. Although the literature suggests that deviations in the experimentally measured equilibrium concentrations may prevent the model from fully fitting the entire reaction range,^42^ the fitted mass changes within the 0–8 s range exhibit a good correlation. In the plasma oxidation experiment, the initial mass of the GO nanosheet on the cantilever was 581.74 ± 0.99 fg, and after reaching equilibrium, the mass increased by 19.26 ± 1.33 fg, indicating that the available sites on the GO nanosheet during the oxidation process accounted for 3.3 wt %. For the HI reduction, the initial mass of the GO nanosheet was 183.92 ± 0.27 fg, with a decrease of 6.92 ± 1.54 fg at the equilibrium state, indicating 3.8 wt % available sites. Similarly, the N and B doping reactions reached equilibrium at 10 and 12 s, respectively, following a pseudo-first-order kinetic model with *R*^2^ values of 0.96 and 0.96, respectively (see Figure S12). N doping increased the GO nanosheet mass by 28.43 ± 3.14 fg, indicating 11.1 wt % available sites (relative to an initial GO mass of 255.52 ± 2.49 fg). B doping increased the mass by 6.89 ± 0.44 fg, indicating 4.4 wt % available sites (relative to an initial GO mass of 154.99 ± 0.58 fg).

## Conclusions

This study introduces a new methodology for monitoring, tracking, and optimizing the chemical and electronic properties of graphene oxide. Using remote liquid-phase plasma treatment, we exploited AFM cantilever resonant frequency shifts to track mass changes in GO during chemical tuning. Using Raman spectroscopy, we observed distinct shifts and broadening in D and G bands of GO, indicative of C–C structural alterations and oxidation processes. Subsequent exposure to a HI environment resulted in GO reduction, as shown by recovery patterns in the Raman bands. Crucially, the AFM cantilever resonant frequency provided a means to convert these observations into quantitative mass change data, revealing oxygen mass variations during oxidation and reduction phases. Furthermore, we used plasma treatment to introduce N and B into the GO structure. This doping process was confirmed through both Raman spectroscopy and XPS. We observed that nitrogen substitution induced a lattice compression strain, manifesting as a blue shift in the Raman G band, while boron substitution resulted in longer B–C bonds and lattice strain, leading to a redshift. These findings validate our methodology of using AFM cantilever resonance frequency changes to monitor reversible reactions in nanomaterials and highlight the combined use of in situ Raman spectroscopy and XPS for detailed atomic-level analysis to achieve femtogram-level mass quantification. This approach offers an innovative and versatile tool for precise monitoring and quantification of material changes during chemical modification, with significant implications for future research in nanomaterials.

## Supporting Information Available

Experimental details, materials, and methods; Raman spectra; XPS spectra; and FTIR spectra (PDF)
