# Supplementary material for: Femtogram-Sensitive Cantilever Platform for Dynamic Graphene Oxide Nanosheet Monitoring
Source: Anal Chem. 2024 Sep 9;96(38):15126–33. doi: 10.1021/acs.analchem.4c01714 (PMC11447974; doi:10.1021/acs.analchem.4c01714)
Supplement: Supplementary file 1 — ac4c01714_si_001.pdf [file ac4c01714_si_001.pdf]

Supplementary Materials

Femtogram-Sensitive Cantilever Platform for Dynamic Graphene Oxide Nanosheet Monitoring

Pei-Ying Lin[a,‡], Sheng-Han Cheng[a,‡], Yu-Chieh Hsu[a,‡], David E. Beck[b], Shuchen Hsieh [a,c,d,e,*]

[a]Department of Chemistry, National Sun Yat-sen University, 70 Lien-Hai Rd., Kaohsiung 80424, Taiwan

[b]Oxford Instruments Asylum Research, Inc., 7416 Hollister Ave., Santa Barbara, CA 93117, USA

[c]Regenerative Medicine and Cell Therapy Research Center, Kaohsiung Medical University, 100 Shih-Chuan 1st Rd., Kaohsiung 80708, Taiwan

[d]School of Pharmacy, College of Pharmacy, Kaohsiung Medical University, 100 Shih-Chuan 1st Rd., Kaohsiung 80708, Taiwan

[e]Institute of Aquatic Science and Technology, College of Hydrosphere Science, National Kaohsiung University of Science and Technology 142, Haijhuan Rd., Kaohsiung 81157, Taiwan

*Email: shsieh@faculty.nsysu.edu.tw

Table of Contents

Experimental Section S4

Characterization S4

DPPH test S4

Reaction Kinetic S5

Results and Discussion S6

Table **S1**. The XPS weight (%) and C/O ratio for different GO treated. S6

Table **S2**. The AFM cantilever resonance frequency shift and total mass change in different GO-doped. S6

Figure **S1**. Frequency response of GO during five cycles of reduction-oxidation. S7

Figure **S2**. (a) Calibration curve for ascorbic acid standard in DPPH scavenging activity at 520 nm. (b) In plasma-treated water with and without GO, relative to the standard of ascorbic acid, the levels of free radicals. S7

Figure **S3**. The XPS spectra of the GO、GO-plasma and GO-HI samples (a) C1s (b) O1s. S8

Figure **S4**. Schematic representation of the possible reduction reaction of GO with HI. S8

Figure **S5**. FTIR spectra of GO treated with plasma oxidation or HI reduction. S9

Figure **S6**. LC-MS/MS of GO-HI. S9

Figure **S7**. The XPS spectra of the N-GO and B-GO samples. (a, d) C1s, (b,e) O1s, (c) N1s, and (f) B1s. S10

Figure **S8**. Schematic representation of potential nitrogen doping structures in N-GO. (i) pyridinic N, (ii) pyrrolic N, (iii) graphitic N, (iv) oxidized N. S10

Figure **S9**. Schematic representation of the reaction for B doping in GO. S11

Figure **S10**. Raman spectroscopy results for GO doping with N and B. (a) Changes in N-GO at different times, (b) Changes in G band shifting in N-GO before and after doping, (c) Changes in B-GO at different times, and (d) Changes in G band shifting in B-GO before and after doping. …… S 1 1

Figure **S11**. Mass changes of GO during (a) plasma oxidation and (b) HI reduction at various time intervals. The pseudo-first-order adsorption kinetics are shown in (c) for GO-plasma oxidation and (d) for GO-HI reduction. ………………………………………………………………………………………… S12

Figure **S12**. Changes in GO mass during (a) N doping and (b) B doping at various time intervals. The pseudo-first-order adsorption kinetics are depicted in (c) for GO-N doping and (d) for GO-B doping.……………………………………………………………………………………………………… S12

References ……………………………………………………………………………………………………… S13

Experimental Section

Characterization

Place the GO nanosheet aqueous solution in a clean 3 mL glass vial and treat the solution directly with various oxidation, reduction, or doping treatments. The reaction time for each treatment should be adjusted according to the final measurement time of the AFM. The oxidation of GO was performed using plasma treatment, while the reduction reaction involved mixing 10 μL of 5 mM HI with 10 μL of GO. For doping with N, or B, 1.6 M NH_3, or 0.8 M H_3BO_3 were added respectively, and the doping reactions were carried out under plasma conditions. The treated GO sample solutions were then deposited onto clean gold substrates for XPS measurements. The XPS data, which provide the elemental ratios, were used to estimate the relative changes in element mass for the AFM cantilever frequency analysis. Elemental analysis was conducted using X-ray photoelectron spectroscopy (XPS), employing a ULVAC-PHI, PHI Quantera II system equipped with a monochromatic Mg Kα X-ray radiation source. Samples were prepared by depositing variously treated GO sample solutions onto clean gold substrates at room temperature.

Raman spectroscopy was used to qualitatively characterize the molecular vibrations of GO after various oxidation, reduction, and doping treatments. A 10 μL of the GO nanosheet aqueous solution was placed on a glass slide, and the spectral changes were monitored in situ using the plasma process method. Oxidation was carried out through plasma treatment, while reduction involved mixing 10 μL of 5 mM HI with GO. For doping with N, or B, 1.6 M NH_3, or 0.8 M H_3BO_3 were added respectively, and the doping reactions were conducted under plasma conditions. The Raman experiment was conducted on a Raman microscope system (WiTec alpha 300R) using a 532 nm laser. The incident laser power was maintained at 13 mW. A holographic grating (1800 lines/mm) and a back-illuminated CCD detector with 1024×127 pixels were employed. The data was recorded with an accumulation time of 2 seconds, with measurements taken every 2 seconds.

DPPH test

The relative free radical performance in water was assessed using DPPH (2,2-diphenyl-1-

picrylhydrazyl) assay. A 200 μM DPPH solution was prepared in methanol. Then, 100 μL of this DPPH solution was mixed with different concentrations (0, 2.5, 5, 10, 20, 40, 50 μM) of vitamin C standard solution (in a 1:1 volume ratio) and allowed to react for 5 minutes. The change in absorbance at the UV wavelength of 520 nm was measured for DPPH. A standard curve was generated by plotting the absorbance intensity change of vitamin C against time. Next, water samples with and without GO were treated with plasma. The water solutions were mixed with 200 μM DPPH (in a 1:1 volume ratio) and allowed to react for 5 minutes. The change in absorbance intensity at the UV wavelength of 520 nm was recorded. The absorbance values obtained were then used to estimate the free radical content by fitting them to the standard curve. This estimated content is expressed relative to the concentration of vitamin C.

Reaction Kinetic

The pseudo-first-order kinetics were observed using the pseudo-first-order kinetic reaction, originally proposed by Lagergren [1]. Generally, the form proposed by Ho and McKay [2] is used: $\ln(me\text{-}mt) = \ln me\text{-}kt$, where mt represents the mass of the element doped at time t, me represents the mass of the element doped at equilibrium, k is the pseudo-first-order rate constant, and t is time.

Results and Discussion

Table S1. The XPS weight (%) and C/O ratio for different GO treated.

Samples	C	O	I	N	B	C/O ratio
GO	54.2	45.8	-			1.19
GO-plasma	54.0	46.0	-			1.17
GO-HI	41.9	33.9	24.3			1.24
N-GO	54.4	45.5		0.1	-	1.20
B-GO	51.5	48.5		-	0.1	1.06

Table S2. The AFM cantilever resonance frequency shift and total mass change in different GO-doped.

Samples		Before	After
GO-plasma	Frequency (Hz)	31857.7 ± 0.2	31856.8 ± 0.1
	mass (fg)	163.2 ± 0.9	167.6 ± 0.4
GO-HI	Frequency (Hz)	31848.0 ± 1.3	31852.5 ± 3.1
	mass (fg)	207.8 ± 5.9	187.1 ± 14.1
N-GO	Frequency (Hz)	31757.7 ± 3.8	31746.7 ± 2.3
	mass (fg)	258.3 ± 7.3	279.5 ± 4.5
B-GO	Frequency (Hz)	31842.9 ± 0.2	31834.1 ± 2.5
	mass (fg)	236.6 ± 0.5	260.9 ± 7.0

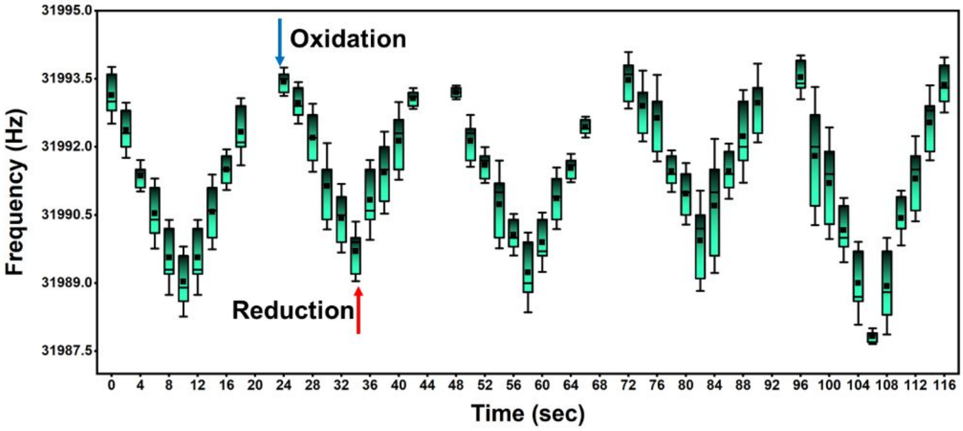

Figure S1. Frequency response of GO during five cycles of reduction-oxidation.

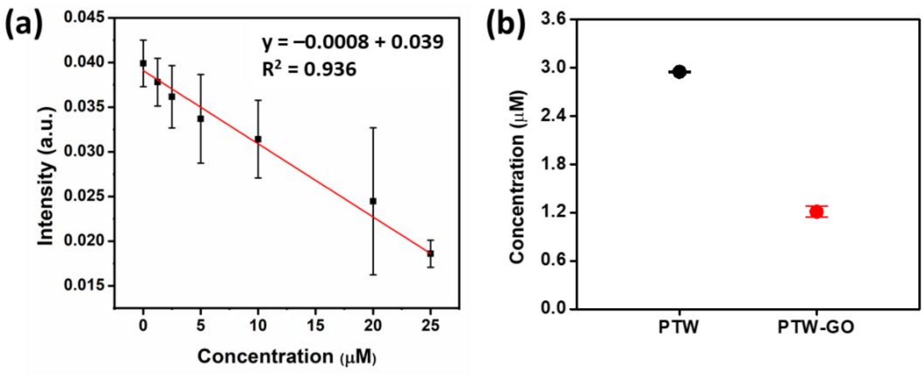

Figure S2. (a) Calibration curve for ascorbic acid standard in DPPH scavenging activity at 520 nm. (b) In plasma-treated water with and without GO, relative to the standard of ascorbic acid, the levels of free radicals.

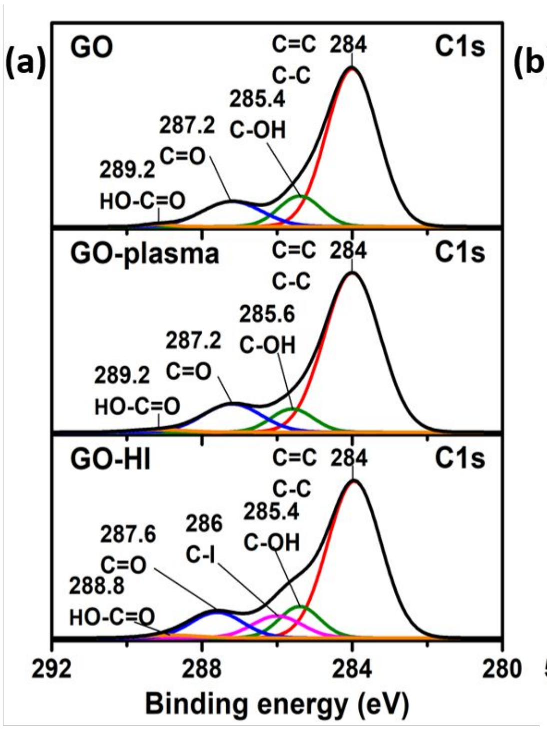

Figure S3. The XPS spectra of the GO、GO-plasma and GO-HI samples (a) C1s (b) O1s.

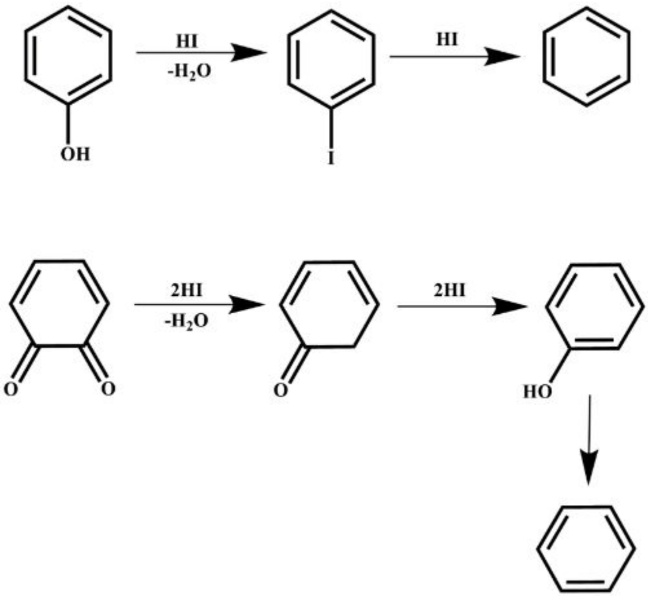

Figure S4. Schematic representation of the possible reduction reaction of GO with HI.

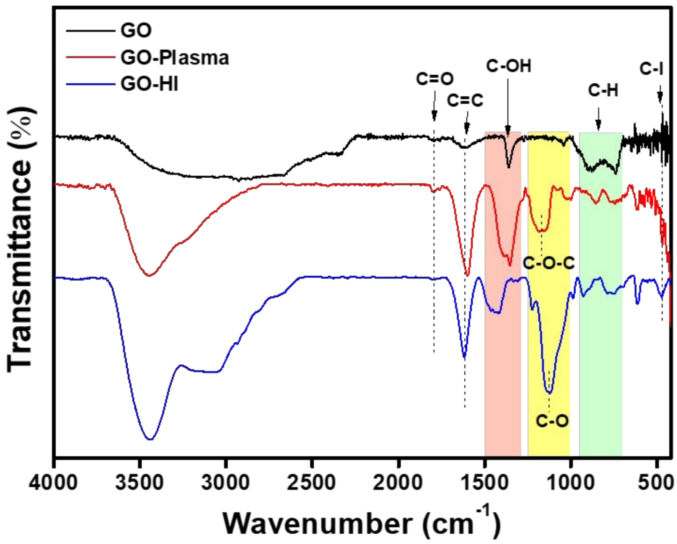

Figure S5. FTIR spectra of GO treated with plasma oxidation or HI reduction.

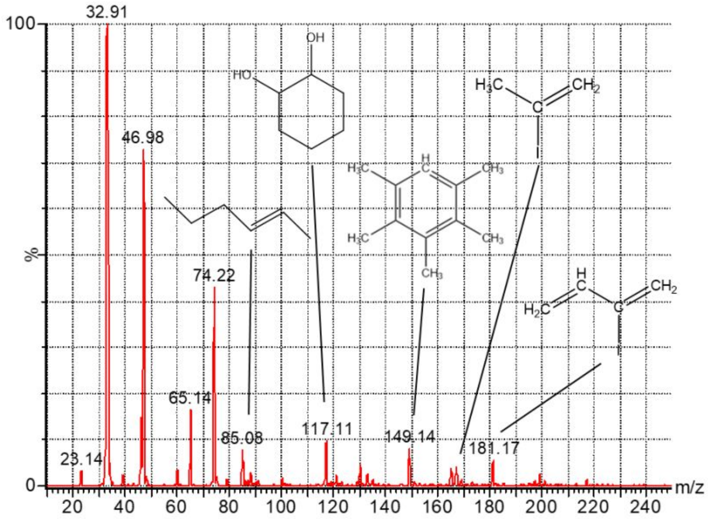

Figure S6. LC-MS/MS of GO-HI.

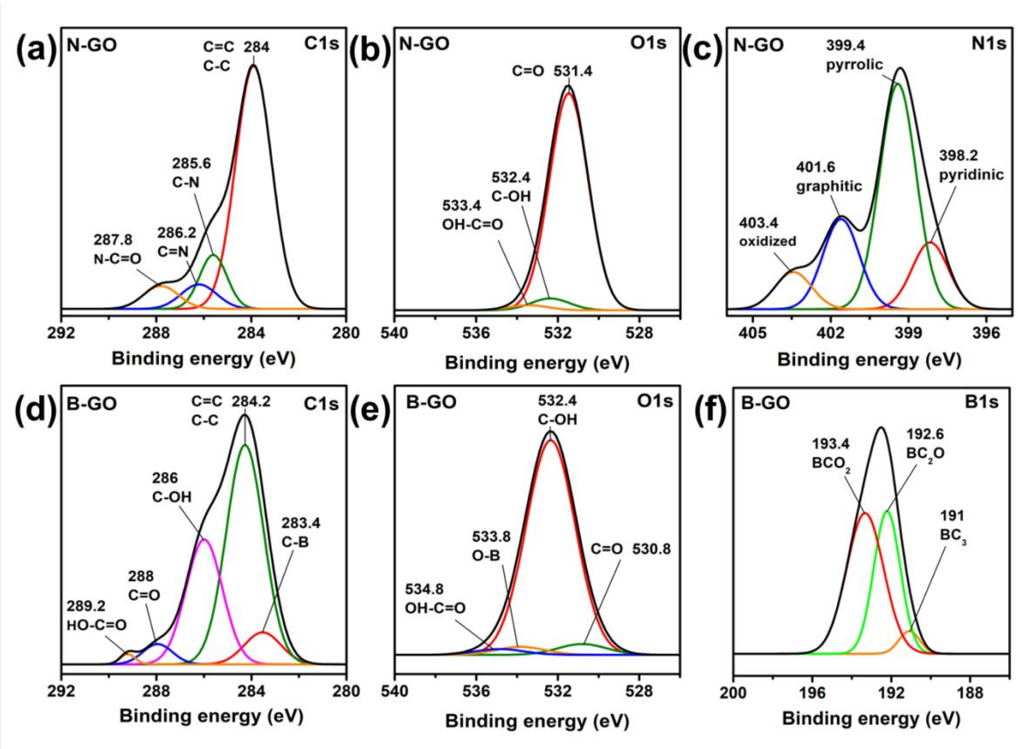

Figure S7. The XPS spectra of the N-GO and B-GO samples. (a, d) C1s, (b,e) O1s, (c) N1s, and (f) B1s.

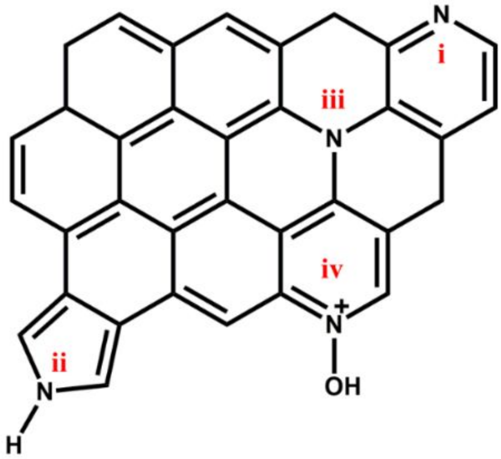

Figure S8. Schematic representation of potential nitrogen doping structures in N-GO. (i) pyridinic N, (ii) pyrrolic N, (iii) graphitic N, (iv) oxidized N.

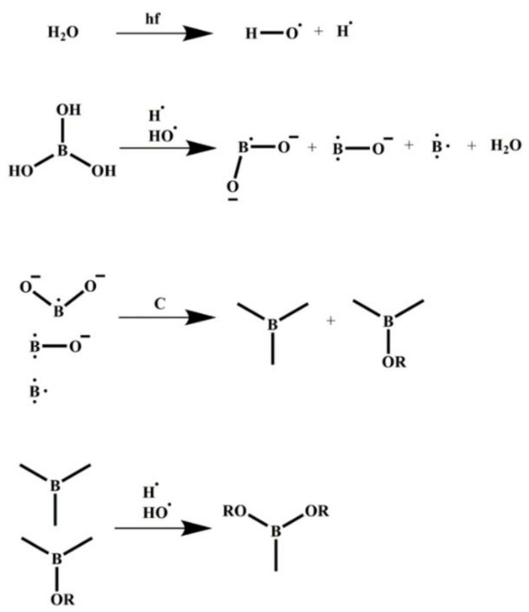

Figure S9. Schematic representation of the reaction for B doping in GO.

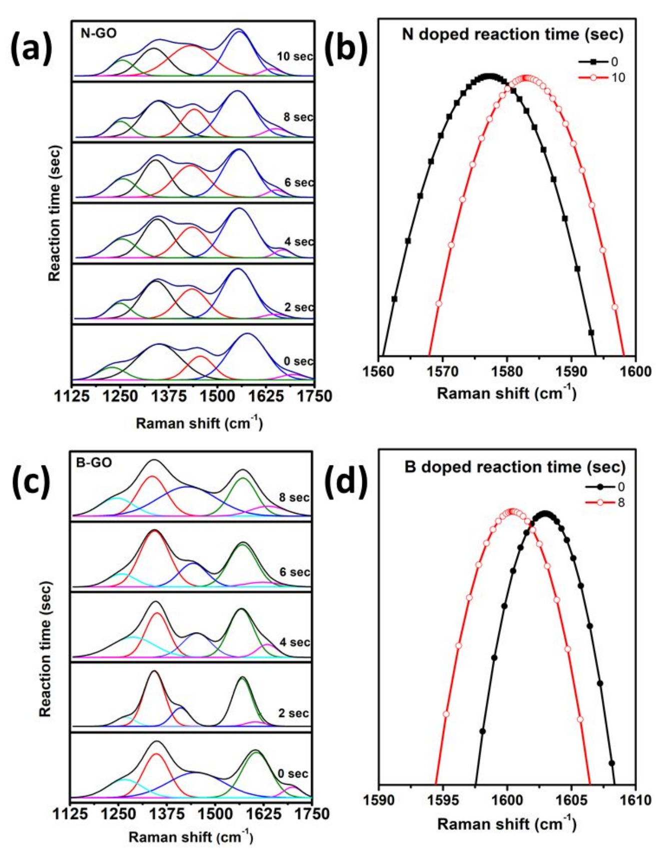

Figure S10. Raman spectroscopy results for GO doping with N and B. (a) Changes in N-GO at different times, (b) Changes in G band shifting in N-GO before and after doping, (c) Changes in B-GO at different times, and (d) Changes in G band shifting in B-GO before and after doping.

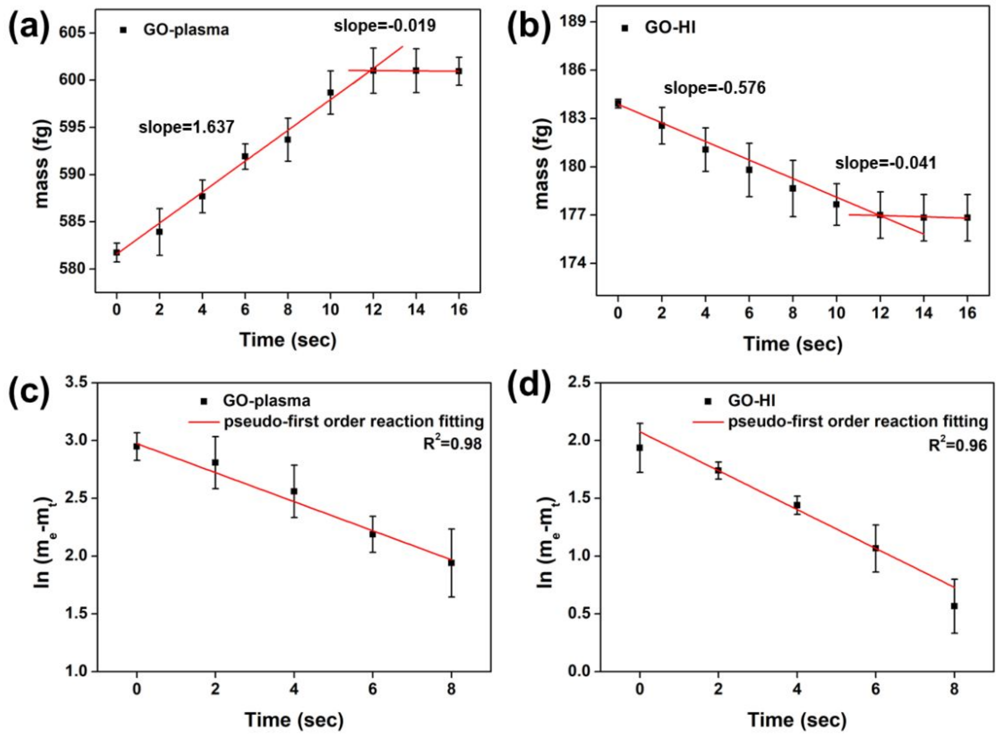

Figure S11. Mass changes of GO during (a) plasma oxidation and (b) HI reduction at various time intervals. The pseudo-first-order adsorption kinetics are shown in (c) for GO-plasma oxidation and (d) for GO-HI reduction.

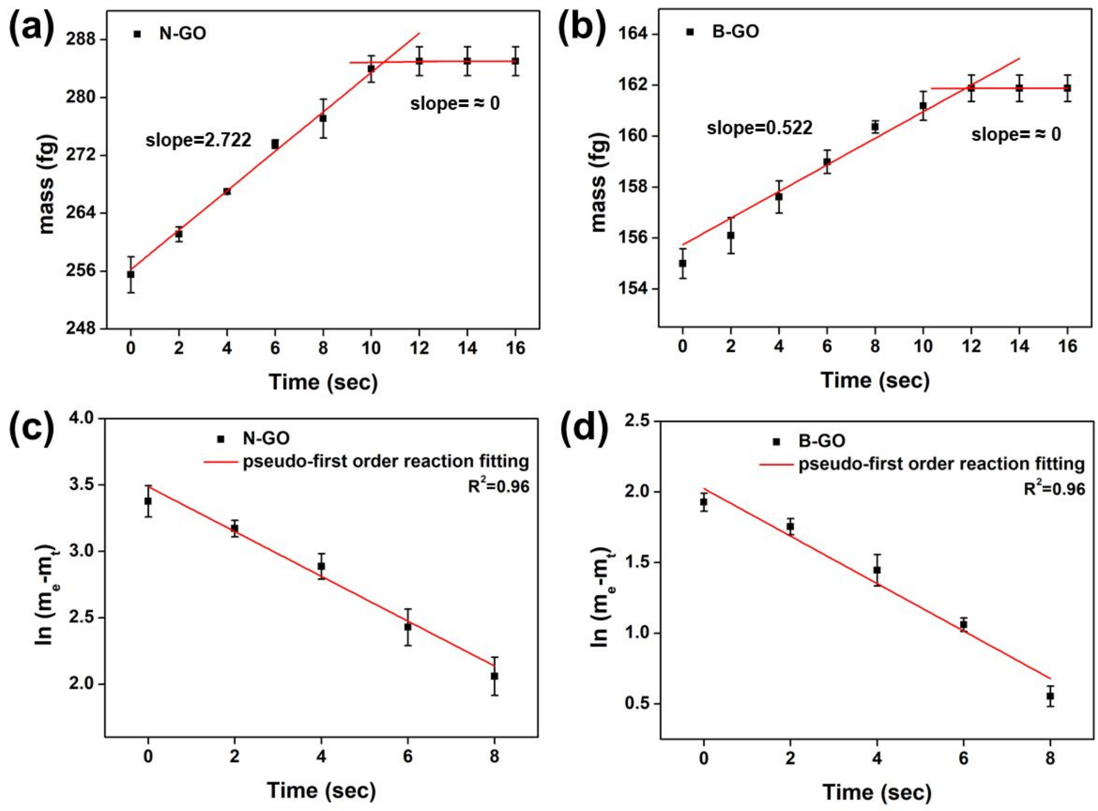

Figure S12. Changes in GO mass during (a) N doping and (b) B doping at various time intervals. The

pseudo-first-order adsorption kinetics are depicted in (c) for GO-N doping and (d) for GO-B doping.

References

[1] Lagergren, S. Zur Theorie der sogenannten Adsorption gelöster Stoffe. *Zeitschrift für Chemie und Industrie der Kolloide*, **1898**, *2*(15), 1-39, DOI: 10.1007/BF01501332

[2] Ho, Y. S., and Gordon M. Pseudo-second order model for sorption processes. *Process Biochem.* 1999, *34*(5), 451-465, DOI: 10.1016/S0032-9592(98)00112-5
